# Replacing non-biomedical concepts improves embedding of biomedical concepts

**DOI:** 10.1371/journal.pone.0322498

**Published:** 2025-05-05

**Authors:** Enock Niyonkuru, Mauricio Soto Gomez, Elena Casarighi, Stephan Antogiovanni, Hannah Blau, Justin T. Reese, Giorgio Valentini, Peter N. Robinson

**Affiliations:** 1 The Jackson Laboratory for Genomic Medicine, Farmington, Connecticut, United States of America; 2 Trinity College, Hartford, Connecticut, United States of America; 3 AnacletoLab, Computer Science Department, Dipartimento di Informatica, Università degli Studi di Milano, Milan, Italy; 4 Division of Environmental Genomics and Systems Biology, Lawrence Berkeley National Laboratory, Berkeley, California, United States of America; 5 Computer Science Department, Aalto University, Espoo, Finland; 6 Berlin Institute of Health at Charité – Universitätsmedizin Berlin, Berlin, Germany; European Commission, ITALY

## Abstract

Embeddings are semantically meaningful representations of words in a vector space, commonly used to enhance downstream machine learning applications. Traditional biomedical embedding techniques often replace all synonymous words representing biological or medical concepts with a unique token, ensuring consistent representation and improving embedding quality. However, the potential impact of replacing non-biomedical concept synonyms has received less attention. Embedding approaches often employ concept replacement to replace concepts that span multiple words, such as non-small-cell lung carcinoma, with a single concept identifier (e.g., D002289). Also, all synonyms of each concept are merged into the same identifier. Here, we additionally leveraged WordNet to identify and replace sets of non-biomedical synonyms with their most common representatives. This combined approach aimed to reduce embedding noise from non-biomedical terms while preserving the integrity of biomedical concept representations. We applied this method to 1,055 biomedical concept sets representing molecular signatures or medical categories and assessed the mean pairwise distance of embeddings with and without non-biomedical synonym replacement. A smaller mean pairwise distance was interpreted as greater intra-cluster coherence and higher embedding quality. Embeddings were generated using the Word2Vec algorithm applied to a corpus of 10 million PubMed abstracts. Our results demonstrate that the addition of non-biomedical synonym replacement reduced the mean intra-cluster distance by an average of 8%, suggesting that this complementary approach enhances embedding quality. Future work will assess its applicability to other embedding techniques and downstream tasks. Python code implementing this method is provided under an open-source license.

## 1 Introduction

Word embeddings are a cornerstone of natural language processing (NLP), enabling machines to represent words as dense vectors in a continuous space where semantic relationships between words are captured by their proximity in that space. One of the most prominent models for generating embeddings is Word2Vec, a two-layer neural network that learns word representations by predicting the context in which a word appears [1]. By analyzing large text corpora, Word2Vec can generate embeddings that reflect linguistic patterns and relationships, supporting tasks such as text classification, clustering, and information retrieval.

Word embeddings are a cornerstone of natural language processing (NLP), enabling machines to represent words as dense vectors in a continuous space where semantic relationships between words are captured by their proximity in that space. One of the most prominent models for generating embeddings is Word2Vec, a two-layer neural network that learns word representations by predicting the context in which a word appears [1]. By analyzing large text corpora, Word2Vec can generate embeddings that reflect linguistic patterns and relationships, supporting tasks such as text classification, clustering, and information retrieval.

The core principle behind Word2Vec is distributional semantics, often summarized by the phrase “the company it keeps” [2]. This means that words occurring in similar contexts tend to have similar meanings and, therefore, similar vector representations. The model operates by training on a corpus of words and their contexts, adjusting the network’s parameters to maximize the likelihood of predicting surrounding words within a defined window size. The resulting vectors capture the syntactic and semantic properties of words, clustering similar terms together in the embedding space [1].

However, while Word2Vec effectively handles individual tokens, it faces limitations when applied to specialized domains such as biomedicine, where multi-word expressions and complex terminologies are prevalent. For example, the phrase “bronchopulmonary dysplasia” represents a single medical condition but would be treated as two unrelated tokens by Word2Vec, leading to fragmented embeddings that fail to capture the full semantic meaning. To address this, recent concept-replacement approaches consolidate multi-word biomedical terms into single tokens or identifiers, allowing embeddings to represent medical concepts more cohesively [3].

Concept replacement has been successfully implemented in biomedical NLP using tools such as the Narrative Information Linear Extraction (NILE) system, which maps terms to the Systematized Nomenclature of Medicine-Clinical Terms (SNOMED-CT), and PubTator, which annotates text with biomedical entities using identifiers from the Medical Subject Headings (MeSH) and other ontologies [4,5]. By replacing synonymous expressions (e.g., “Myocardial Infarction”, “Heart Attack”) with a shared identifier (e.g., MeSH D009203), these approaches standardize terminology, enhance embedding performance, and facilitate downstream tasks such as entity recognition and relationship extraction.

The benefits of concept replacement extend beyond simply collapsing multi-word terms. By grouping synonymous but distinct expressions under a single token, embeddings are trained on more diverse and informative contexts, enhancing the generalization and quality of word representations. This results in reduced intra-cluster distances for embeddings of related terms and clearer separation between unrelated terms. Additionally, the reduced vocabulary size can accelerate training convergence and improve model efficiency. Prior studies have demonstrated that biomedical concept replacement significantly improves embeddings for domain-specific tasks [3].

While these advancements have enhanced embeddings for biomedical terms, existing efforts have largely overlooked the impact of non-biomedical synonyms. In biomedical literature, non-biomedical terms (such as general descriptors or measurements) frequently appear alongside technical terms, contributing to noise in embeddings. We hypothesize that replacing non-biomedical synonyms in the same manner as biomedical terms could further improve embedding quality by reducing variability in the contextual environment.

**Fig 1:**
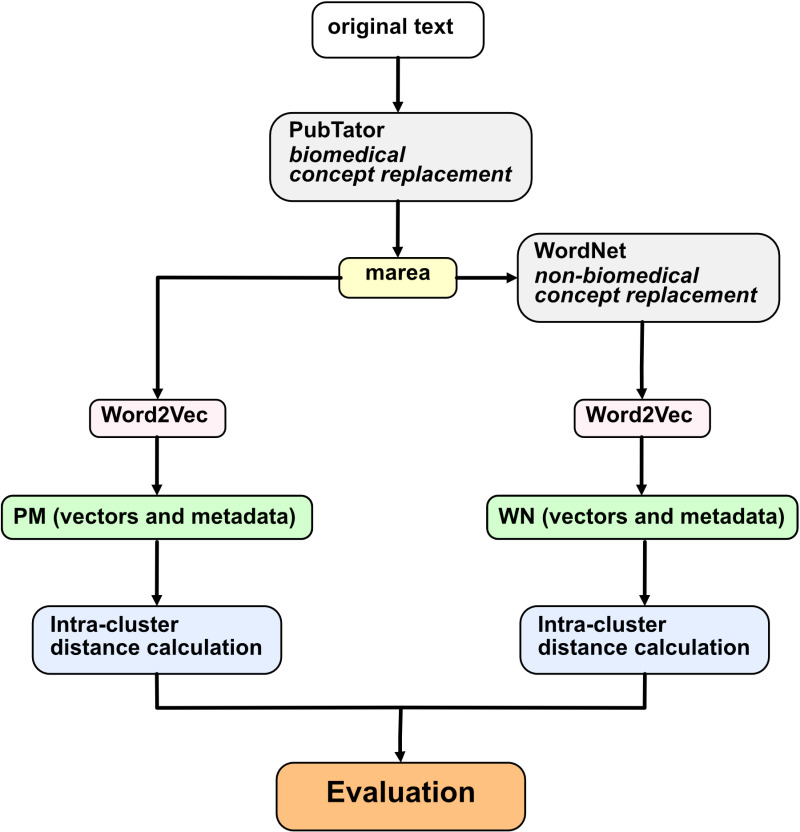
Schematic of the approach: This schematic illustrates the entire workflow of the project. The process begins with initial text preprocessing using marea software to obtain the PM corpus [6]. The PM corpus is then processed through non-biomedical concept replacement, resulting in the WN corpus; to fairly assess the concept replacement proposal, both the PM and WN corpora are embedded using the same text-embedding algorithm (Word2Vec in our experiments - due to its broad usage and relative simplicity), and pairwise distances between sets of related biomedical concepts in the embedded PM corpus are compared to those in the embedded WN corpus.

In this study, we propose a simple yet effective heuristic for non-biomedical synonym replacement aimed at refining embeddings for biomedical texts. Building on the foundation of existing biomedical synonym replacement approaches, we apply our method to over 30 million PubMed abstracts and titles. Our analysis of 1055 gene sets demonstrates that replacing non-biomedical synonyms leads to an average improvement of 8% in embedding performance. The process is summarized in Fig 1 The results show that embedding homophily—reflected by tighter intra-cluster distances—can be enhanced not only by consolidating biomedical concepts but also by refining the non-biomedical terms that shape their contextual embeddings.

This work contributes to the broader field of biomedical NLP by addressing a previously unexplored gap: the role of non-biomedical synonyms in embedding performance. By extending synonym replacement beyond biomedical terms, this study highlights a scalable strategy to improve embeddings for complex biomedical corpora. Future work will explore the applicability of this approach to advanced models, such as BERT and BioBERT, to further enhance the accuracy and interpretability of biomedical embeddings.

## 2 Materials and methods

### 2.1 Input corpus retrieval and text pre-processing with MAREA

The corpus used to test our proposal consists of 10,584,195 abstracts and titles published between January 2010 and November 2020 and available in PubMed. They were downloaded from the National Center for Biotechnology Information (NCBI) ‘s FTP site using MAREA, a software tool designed to automate the retrieval and parsing of PubMed metadata, including the extraction of PubMed IDs and publication dates [6]. Marea is freely available at https://github.com/TheJacksonLaboratory/marea.

**Fig 2:**
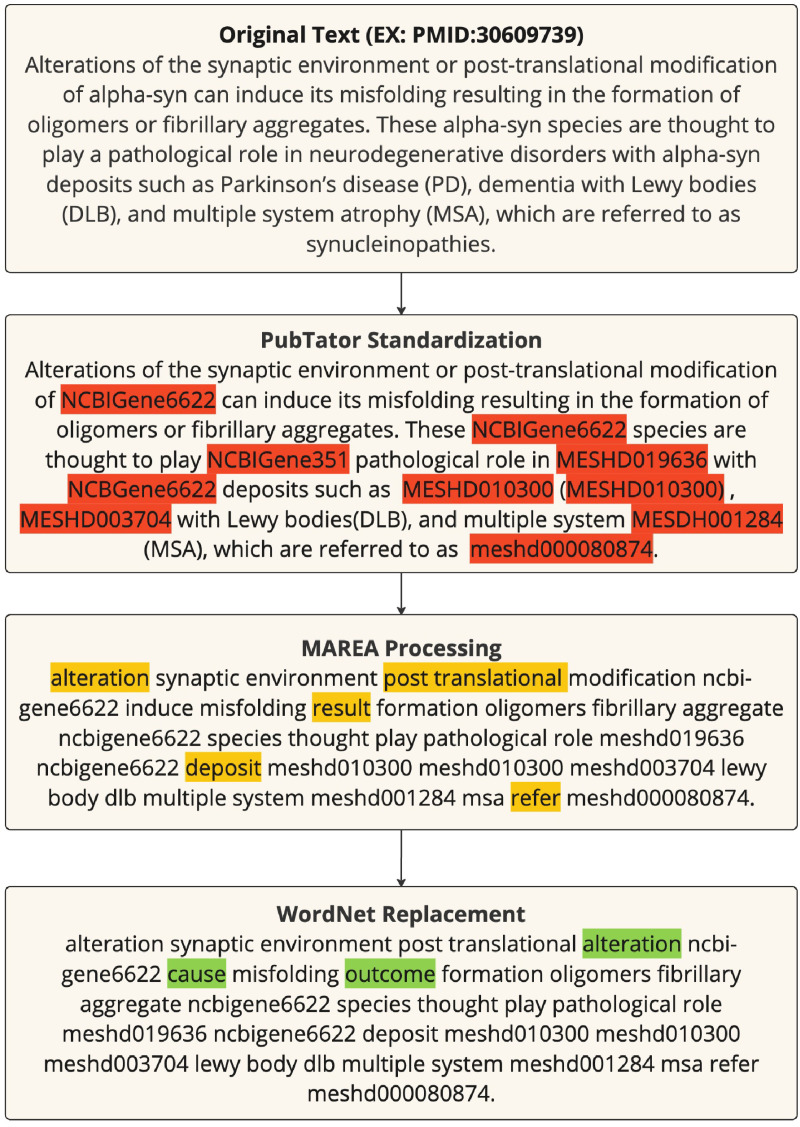
Text Transformation Pipeline: An example of the multi-stage text transformation pipeline applied to a sample abstract (PMID: 30609739). The process begins with the original text, followed by biomedical entity recognition and standardization using PubTator, which replaces medical terms and their synonyms with standardized identifiers (e.g., MeSH IDs). The text is then processed by MAREA, which simplifies and prepares it for machine learning by retaining standardized biomedical terms and ensuring consistent tokenization. In the final stage, non-biomedical synonyms are replaced using WordNet to further refine the embeddings. This figure illustrates the transformation applied across 30 million abstracts.

Marea was also employed to perform automatic text pre-processing and standardization of biomedical concepts across the corpus, reducing noise and improving consistency in preparation for embedding. As illustrated in Fig 2, the pre-processing pipeline begins with the application of PubTator Central, which replaces single- or multi-word concepts and synonyms with unique concept identifiers, such as MeSH IDs. This step is essential for handling multi-word noun phrases representing diseases, chemicals, or other biomedical entities, ensuring that all synonymous terms are treated uniformly during downstream processing. It is important to note that while PubTator effectively standardizes many biomedical entities, such as genes, diseases, and chemicals, its coverage may vary depending on the specific entity type and the availability of mappings within controlled vocabularies like MeSH, NCBI Gene, and Disease Ontology [5]. As a result, certain terms, such as “Lewy bodies (DLB)” or “multiple system atrophy (MSA),” may not always be directly standardized by PubTator.

Following biomedical concept replacement, marea further streamlines the text by removing extraneous information, including punctuation, numerical values, and stop words. The vocabulary is further reduced through lemmatization, enhancing the focus on meaningful terms while preparing the corpus for subsequent embedding tasks.

### 2.2 Replacement of non-biomedical words by their WordNet synonym

The hypothesis of this research is that the replacement of sets of highly related non-biomedical concepts by their common synonym will increase the ability of an embedding algorithm, e.g., Word2Vec, to place related biomedical concepts close to each other in vector space.

To identify synonyms of common words, we queried WordNet, a lexical database of English that groups nouns, verbs, adjectives, and adverbs into sets of cognitive synonyms (synsets), each expressing a distinct concept [7]. Words are interlinked by conceptual-semantic and lexical relations (https://wordnet.princeton.edu/).

The replacement algorithm we devised starts by identifying the set of non-biomedical concepts (words) to be replaced. This choice is based on the overall frequency, f(w), of each token, *w*, in the corpus (multiple occurrences in one abstract were counted multiple times).

In particular, we reasoned that words frequently appearing in the corpus might be important and should not be replaced.

Therefore, the algorithm starts by selecting relatively infrequent words, i.e., words with f(w)<τ, being τ a user-set replacement threshold, as candidates for replacement. These candidates are inserted in a “replacement set”, R. We experimentally chose τ as the mean of the overall frequency for all tokens in the corpus in Section 2.3.2.

*R* contains infrequent words that can be clustered into two groups based on the overall frequency of their synonyms: (1) infrequent words whose synonyms are frequent in the corpus (e.g., loquacious, obfuscate) carry generic meanings and can therefore be replaced by their most frequent synonym; (2) infrequent words whose synonyms are also infrequent in the corpus (e.g., peregrinate, recondite). Our heuristic posits that such words are likely to have highly-specific meanings providing detailed, and possibly discriminatory, information and should therefore be retained.

Based on these considerations, the replacement algorithm uses WordNet to identify the synset ($S_{w}$) of each w ∈ R ; next it selects the synonym of w with the highest overall-frequency in the corpus, $s_{\max}$, and stores it in a dictionary, S, mapping the word w to $s_{\max}$, i.e.,

$S[w]\;=\;s_{\max}={argmax}_{x\;\in \;S_{w}}\;f(s)$

**Fig 3:**
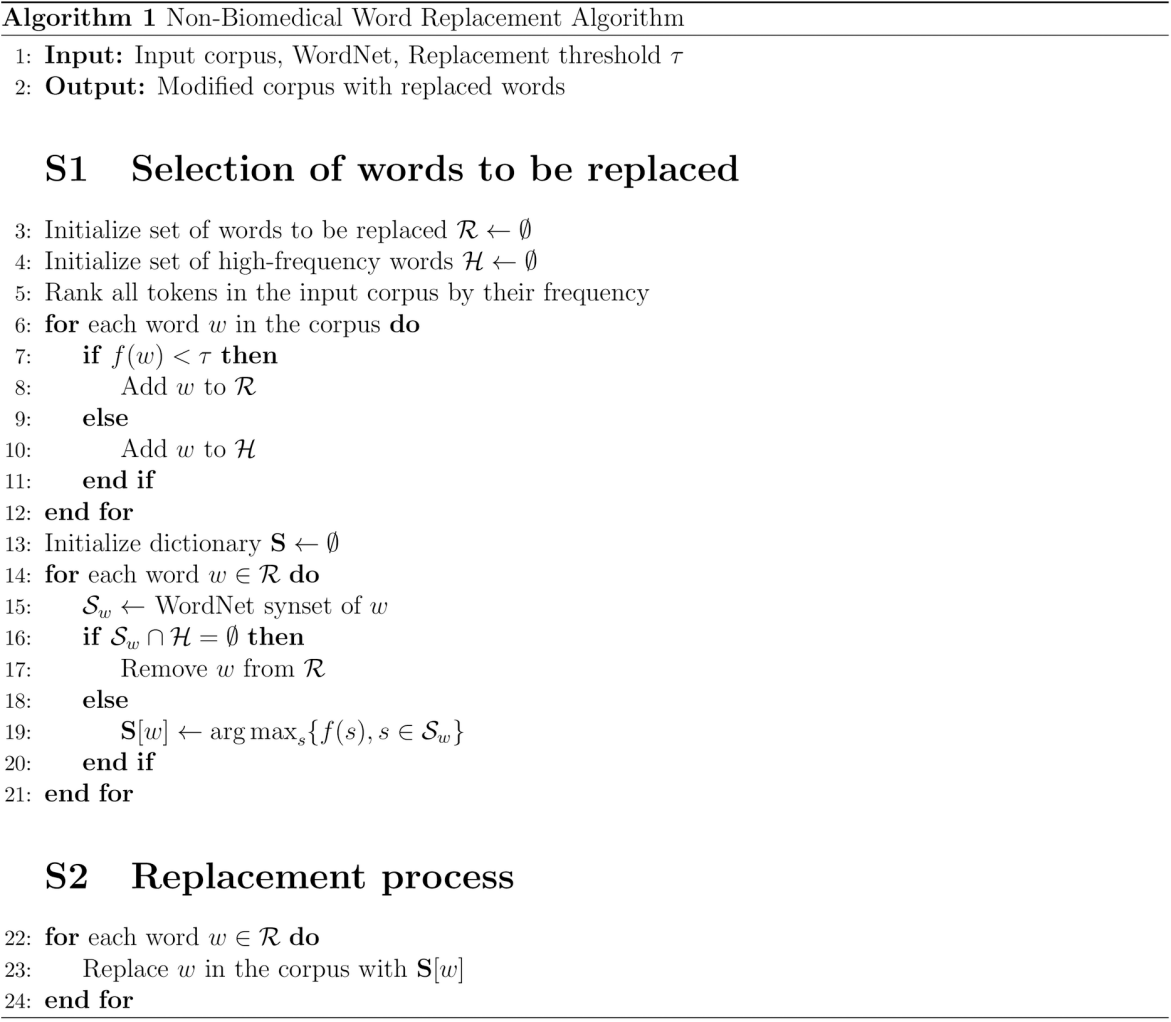
Non-biomedical word replacement algorithm: This algorithm outlines the process for replacing non-biomedical words in a corpus using WordNet.

Words whose synonyms are all infrequent in the corpus are easily recognized through the dictionary because if the frequency of all synonyms of some word w is below the threshold (f(s< τ), Then clearly the frequency of the most common synonym is below the threshold. They are not replaced and removed from R. Each other word w ∈ R is instead replaced by its most frequent synonym S[w]. The algorithm pseudo code is available in Fig 3, and a practical example of the replacement process is reported in Fig 3 with a sample text we created to illustrate the algorithm implementation.

**Fig 4:**
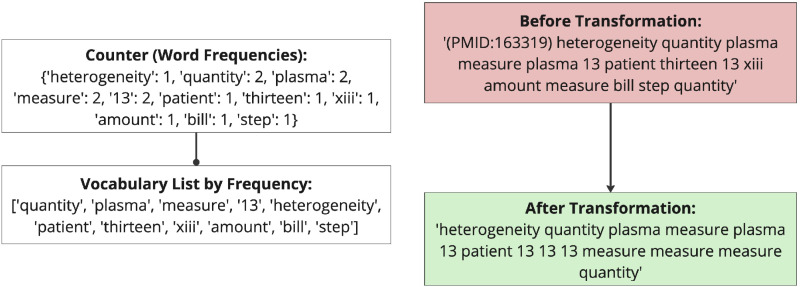
Illustration of the text transformation process before and after synonym replacement: The process begins with a sample initial text segment (Sample text before synonym replacement’), followed by a word frequency count (‘Counter’). This count generates a ‘Vocabulary List by Frequency’, which informs the final modified text (‘Sample text after synonym replacement’). The procedure exemplifies the algorithm’s systematic approach to replacing non-biomedical tokens.

Fig 4 illustrates the synonym replacement process applied to a sample abstract. The transformation pipeline demonstrates how non-biomedical terms are replaced using their most frequently occurring synonym from WordNet synsets. The figure shows the initial sentence before transformation, the word frequency counter, the ordered vocabulary list by frequency, and the resulting sentence after transformation.

In this example, the terms “amount,” “bill,” and “step” are replaced by the synonym “measure” because they all belong to the same WordNet synset. The synset for “measure” includes: [*‘measure’, ‘step’, ‘quantity’, ‘amount’, ‘bill’, ‘measurement’, ‘standard’, ‘criterion’, ‘touchstone’, ‘meter’, ‘metre’, ‘cadence’, ‘bar’*]. Since “measure” appears most frequently in the corpus, the algorithm selects it as the main term.

Similarly, the term “13” replaces variations such as “thirteen” and “xiii” as they are part of the same synset: [*‘thirteen’, ‘13’, ‘XIII’, “baker’s dozen”, ‘long dozen’, ‘xiii’*].

The replacement ensures that numerical references are consistently represented by the most dominant form (“13”). The result is a more uniform representation of non-biomedical terms, reducing variability and enhancing embedding quality by consolidating terms under their most frequently occurring synonym.

### 2.3 Experiments

In the following sections we will refer to datasets processed only by MAREA as PubMed-MAREA, or “PM”; PM datasets further processed to substitute (not-frequent) non-biomedical concepts by their WordNet synonyms will be referred to as “WN”. While the number of unique biomedical concepts did not change between the PM and the WN corpus, there were more unique non-biomedical concepts in PM (3,018,918) than in WN (2,992,978).

We derived embeddings representing the concepts in the input corpora (the 10,579,997 PM or WN abstracts) by adapting the Word2Vec [1] implementation provided by the Gensim library [8]. In particular, we used skip-gram architecture with embedding size 128 (meaning that all concepts in PM and WN were represented as 128-dimensional vectors), window size 10, included words in the vocabulary that appear at least once in the corpus (mincount = 1), and applied a sampling threshold of for downsampling high-frequency words. The initial learning rate was set to 0.03 (alpha = 0.03) and was linearly decreased to a minimum of 0.0001 (min-alpha = 0.0001) during training; we fixed the number of negative samples per positive context word to 5.

#### 2.3.1 Concept sets.

Our assumption is that the quality of embeddings can be assessed by measuring the pairwise distances between the embeddings of related concepts. To evaluate our proposal we therefore identified subsets of related genes and medical concepts prior to performing the testing and validation described in the following section. The sets are available in the project’s GitHub repository. Files containing the sets can be identified by the suffix “-set” in their filenames. https://github.com/TheJacksonLaboratory/wn2vec/tree/main/data.

961 gene subsets were retrieved from the Molecular Signatures Database (MSigDB) [9] (Table 1). In addition, 94 subsets of related medical concepts were retrieved from the Medical subject headings (MeSH) resource [10].

**Table 1:** Comparison of mean interconcept distance for embedding with WordNet synonym replacement (WN) and without (PM). The initial number of unique concepts in the total corpus was 3,018,918. The Table summarizes results for different thresholds (τ) and categories of concept/gene sets (M,B,K,G,P). Columns: τ: Replacement threshold; replaced: Unique Replaced Concepts; Category: M = MeSH, B = Biocarta, K = KEGG, G = GP(bp), P = PID; # sets: Number of concept/gene sets in the categories; #Concepts: number of concept vectors in the category; *WN better: The count and percentage of concept/gene sets for which the mean interconcept distance was smaller for WN than for PM. “Winners” are shown in bold.;* *PM better: Analogous to “WN better” but for PM.*

						Significant		All	
τ	Replaced	% of unique concepts replacement	Category	# sets	# Concepts	*WN better*	*PM better*	*WN better*	*PM better*
0.5·μ	24,294	0.80 %	M	94	2503	**12 (12.8%)**	4 (4.3%)	40 (42.6%)	**48 (51.1%)**
			B	285	1480	**22 (7.7%)**	19 (6.7%)	**145 (50.9%)**	140 (49.1%)
			K	182	4941	32 (17.6%)	21 (11.5%)	90 (49.5%)	**92 (50.5%)**
			G	300	7030	**36 (12.0%)**	31 (10.3%)	**137 (45.7%)**	135 (45.0%)
			P	194	2507	18 (9.3%)	**26 (13.4%)**	96 (49.5%)	**98 (50.5%)**
1∙μ	25,940	0.86%	M	94	2503	**13 (13.8%)**	9 (9.6%)	**46 (48.9%)**	42 (44.7%)
			B	285	1480	**32 (11.2%)**	12 (4.2%)	**154 (54.0%)**	131 (46.0%)
			K	182	4941	**35 (19.2%)**	13 (7.1%)	**112 (61.5%)**	70 (38.5%)
			G	300	7030	**36 (12.0%)**	27 (9.0%)	**147 (49.0%)**	125 (41.7%)
			P	194	2507	**29 (14.9%)**	5 (2.6%)	**115 (59.3%)**	79 (40.7%)
2∙μ	27,317	0.90%	M	94	2503	**13 (13.8%)**	6 (6.4%)	**49 (52.1%)**	39 (41.5%)
			B	285	1480	22 (7.7%)	**28 (9.8%)**	**153 (53.7%)**	132 (46.3%)
			K	182	4941	**37 (20.3%)**	24 (13.2%)	**105 (57.7%)**	77 (42.3%)
			G	300	7030	34 (11.3%)	**37 (12.3%)**	**148 (49.3%)**	124 (41.3%)
			P	194	2507	**27 (13.9%)**	22 (11.3%)	**105 (54.1%)**	89 (45.9%)
4∙μ	28,418	0.94%	M	94	2503	**14 (5.1%)**	7 (2.5%)	**53 (19.2%)**	35 (12.7%)
			B	285	1480	26 (8.8%)	**36 (12.1%)**	121 (40.7%)	**164 (55.2%)**
			K	182	4941	**37 (11.6%)**	21 (6.6%)	**96 (30.1%)**	86 (27.0%)
			G	300	7030	**33 (9.7%)**	32 (9.4%)	**144 (42.4%)**	128 (37.6%)
			P	194	2507	18 (5.0%)	**34 (9.4%)**	**89 (24.6%)**	105 (29.0%)

Concept subsets were deleted if they contained less than 5 concepts that were represented in the test (PM or WN) corpus. The minimum number of concepts in a set to be considered was fixed to 5 concepts under the assumption that larger sets would have less semantic focus.

For example, if a gene set had 100 genes but, in our corpus, only 3 genes belonging to the gene set were mentioned, then that gene set was deleted.

#### 2.3.2 Testing and validation.

**Fig 5:**
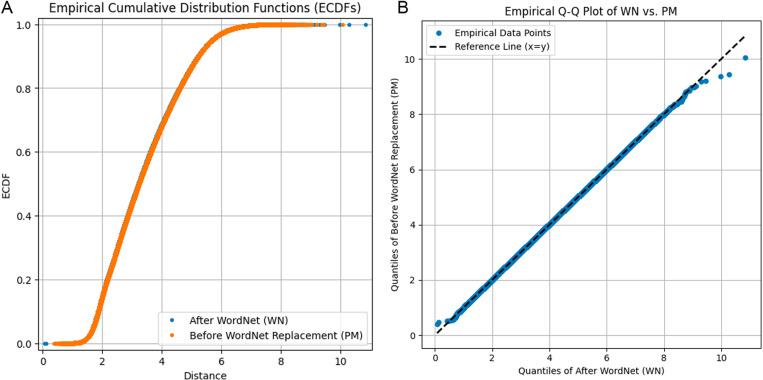
Comparative analysis of WordNet replacement impact on data distribution. Figure (a) presents the Empirical Cumulative Distribution Functions (ECDFs), showcasing the cumulative frequency distribution before and after WordNet replacement, while Figure (b) illustrates the corresponding Empirical Q-Q Plot, detailing the quantile comparison between the original and the WordNet-replaced datasets. The close alignment of data points with the reference line in the Q-Q Plot and the overlap of the ECDF curves suggest minimal distributional deviation post-replacement.

We first checked that the scale and distribution of PM and WN vectorial space did not change. To this aim, we randomly sampled 1M vector-pairs in each dataset. We then calculated the distance between pairs of vectors and then plot the Empirical Cumulative Distribution Function (ECDFs) and Empirical Q-Q Plot of the computed distances (Fig 5). We visually verified that the two distributions had only slight differences.

Next, we analyzed the embedded representations obtained after PM and WN processing by focusing on individual subsets, X (Section 2.3.1), and employing cosine similarity to evaluate all pairwise distances among the embedded concepts within X’s representation post-PM processing versus post-WN processing. We then used the t-test to compare the pairwise-distances computed within the PM subset against those within the WN subset.

**Fig 6:**
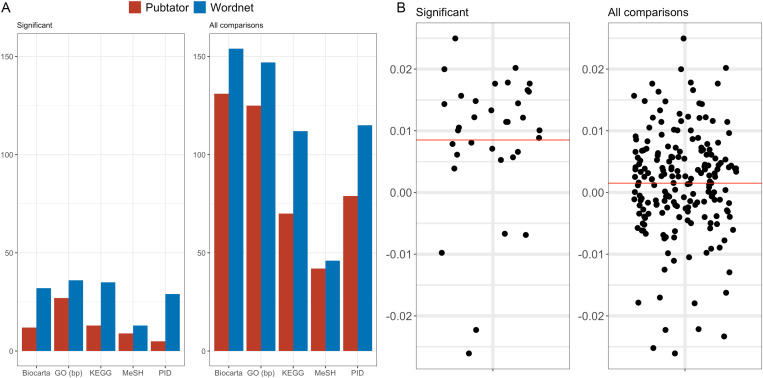
Comparative analysis of WN and PM methodologies: Figure (a) displays the bar chart comparing WN and PM across five distinct concept sets (Methods), highlighting the number of concept sets where the cluster mean distance is significantly lower, indicative of superior embeddings. ‘Significant’ designates those with a statistically significant difference in cluster mean distances (p<0.05), while ‘All Comparisons’ encompasses the entire dataset. Figure (b) illustrates the spread of mean distances within the PID Gene Sets, detailing the variance and central tendency across 194 gene sets. ‘Significant’ encompasses gene sets with notable mean distance variations between ‘PM’ and ‘WN’ (p<0.05), and ‘All comparisons’ includes all evaluated gene sets.

We observed that the application of our replacement strategy leads to an intra-cluster mean distance that is smaller than for the non-replaced data. Indeed, over 1,055 sets of related gene and MeSH concepts sets, we found that, on average, the mean intra-cluster distance was reduced of the 8% - for sets where a significant difference was found, and by the 12% - on the average of all the comparisons (Fig 6).

We also tested different thresholds for replacing non-biomedical terms (i.e., 214, 854, 1708, and the mean value of 427). We found that using the mean value yielded the best results (Table 1). Lower thresholds resulted in fewer words being replaced, while higher thresholds risked losing context by replacing too many words.

In addition, we investigated the impact of different parameters on the performance of our method. We varied the window size number, the higher the window size (i.e., 2, 5, and 10), the stronger the embeddings, and the more the WordNet synonym replacement had an impact on biomedical concept embeddings. The best results were obtained with a window size of 10 (Table 2).

**Table 2:** Comparison of window size for embedding with Wordnet synonym replacement (WN) and without (PM). While Table 1 compared the effects of different values of τ using a window size of 10, this Table shows the results for three different window sizes at a τ=1·μ. Abbreviations are the same as for Table 1.

				Significant		All	
Window Size	Category	# sets	# Concepts	*WN better*	*PM better*	*WN better*	*PM better*
2	MESH	94	2503	9 (9.6%)	**10 (10.6%)**	37 (39.4%)	**51 (54.3%)**
	Biocarta	285	1480	**33 (11.6%)**	30 (10.5%)	**152 (53.3%)**	133 (46.7%)
	KEGG	182	4941	**34 (18.7%)**	20 (11.0%)	**101 (55.5%)**	81 (44.5%)
	GO(bp)	300	7030	39 (13.0%)	**40 (13.3%)**	**144 (48.0%)**	128 (42.7%)
	PID	194	2507	**39 (20.1%)**	24 (12.4%)	96 (49.5%)	**98 (50.5%)**
5	MESH	94	2503	**14 (14.9%)**	12 (12.8%)	43 (45.7%)	**45 (47.9%)**
	Biocarta	285	1480	**27 (9.5%)**	21 (7.4%)	**152 (53.3%)**	133 (46.7%)
	KEGG	182	4941	**37 (20.3%)**	17 (9.3%)	**104 (57.1%)**	78 (42.9%)
	GO(bp)	300	7030	**36 (12.0%)**	27 (9.0%)	**144 (48.0%)**	128 (42.7%)
	PID	194	2507	**27 (13.9%)**	20 (10.3%)	**102 (52.6%)**	92 (47.4%)
10	MESH	94	2503	**13 (13.8%)**	9 (9.6%)	**46 (48.9%)**	42 (44.7%)
	Biocarta	285	1480	**32 (11.2%)**	12 (4.2%)	**154 (54.0%)**	131 (46.0%)
	KEGG	182	4941	**35 (19.2%)**	13 (7.1%)	**112 (61.5%)**	70 (38.5%)
	GO(bp)	300	7030	**36 (12.0%)**	27 (9.0%)	**147 (49.0%)**	125 (41.7%)
	PID	194	2507	**29 (14.9%)**	5 (2.6%)	**115 (59.3%)**	79 (40.7%)

“WN better” means that WN embedding produced concept vectors that were closer to each other than PM, and “PM better” means that the PM produced vectors that were closer to each other. Data are shown for statistically significant (Sig) differences and for all comparisons (All). The “winner“ in each comparison is shown in bold. For significant differences, WN was superior in 14 of 16 cases; For all differences, WN was superior in 13 of 16 cases. The analysis was performed at a window size of 10.0.

## 3 Discussion

### 3.1 Interpretation

This study demonstrates that replacing non-biomedical concepts with their synonyms enhances the quality of biomedical embeddings. By applying the Word2Vec algorithm to over 30 million PubMed abstracts and titles, we evaluated 1,055 gene sets and observed an 8% improvement in embedding performance. This improvement suggests that synonym replacement of non-biomedical terms enhances homophily in the embedding space, resulting in reduced intra-cluster distances and clearer separations between related and unrelated biomedical concepts. Homophily in this context reflects the natural tendency for embeddings of related biomedical terms to cluster together, fostering greater semantic coherence across the vector space.

The experiments conducted with varying thresholds revealed that larger datasets generally yielded better embedding performance. Using a replacement threshold equal to the mean frequency of concepts in the overall corpus emerged as the most effective strategy. Excessive synonym replacement, driven by overly high thresholds, diminished the quality of embeddings; this may be related to inadvertent replacement of biomedical terms. Additionally, the results indicated that larger window sizes in the Word2Vec algorithm led to tighter intra-cluster distances, suggesting that expanding the context window enables embeddings to capture richer contextual relationships and further improve clustering after synonym replacement.

### 3.2 Limitations

Despite the improvements demonstrated in this study, several limitations must be acknowledged. First, our approach is constrained by its reliance on English-language resources such as WordNet and PubTator, and thus is currently only available for English. The absence of robust synonym databases in other languages poses a significant barrier to extending this method to other languages.

Additionally, the study employed Word2Vec, a widely used but relatively simple embedding algorithm, to evaluate the effects of synonym replacement. While Word2Vec provides a solid foundation for demonstrating the utility of non-biomedical synonym replacement, the performance of more advanced models, such as transformer-based architectures, may yield different results. Transformer models were developed for NLP problems to address long-range dependencies through the attention mechanism [11]. Large language models (LLMs) are a class of foundation models with billions of parameters trained on language corpora with billions of words to generate human-like language and solve many NLP tasks. Most LLMs use the Transformer architecture [12]. While the non-biomedical synonym replacement strategy presented here could be applied to transformed-based models, the computational costs went beyond the scope of the current pilot study.

Another limitation of this study lies in the variability of biomedical terminology, particularly in patient narratives or informal texts. While the synonym replacement approach effectively standardizes non-biomedical terms, it may not fully capture the contextual variability inherent in less formal biomedical texts. This variability can introduce inconsistencies that reduce the effectiveness of synonym replacement, particularly when dealing with highly specialized or colloquial expressions. Furthermore, certain biomedical terms may lack standardized mappings in existing databases, resulting in incomplete synonym replacement and limiting the overall impact on embedding quality.

### 3.3 Future directions

The findings of this study provide a foundation for future research exploring the integration of synonym replacement strategies into more advanced embedding models. While the current study focused on Word2Vec, the methodology can be extended to transformer-based models such as BERT, BioBERT, and SciBERT, [13] which have demonstrated superior performance in biomedical text processing. Transformer models, with their capacity to capture complex linguistic patterns and polysemy, may benefit significantly from synonym replacement during the pre-training or fine-tuning phases. This could lead to enhanced contextual embeddings and further improvements in downstream biomedical tasks, including named entity recognition, relation extraction, and document classification.

Moreover, applying synonym replacement strategies to other types of biomedical text, such as clinical trial reports, electronic health records (EHRs), and patient narratives, represents a promising avenue for future research. These text sources frequently contain a mix of biomedical and non-biomedical terminology, and refining embeddings in these contexts could yield significant benefits for clinical decision support systems and predictive modeling. Improved embeddings may enhance the extraction of insights from diverse biomedical datasets, ultimately contributing to advancements in biomedical informatics and precision medicine.

While Word2Vec continues to serve as a lightweight and computationally efficient tool for large-scale corpus analysis, its limitations compared to transformer-based models underscore the need for continued exploration of more sophisticated architectures. Nevertheless, the improvements observed in this study demonstrate that even simple embedding models can benefit from synonym replacement, offering practical enhancements for existing biomedical pipelines that may lack the resources to implement more computationally intensive models. By refining embeddings through synonym replacement, this study addresses a critical gap in biomedical text processing, laying the groundwork for more accurate and meaningful vector representations across various biomedical domains.

## 4 Conclusion

The results of this study highlight the potential of non-biomedical synonym replacement to enhance the quality of biomedical embeddings, offering practical applications across multiple domains of biomedical informatics. By refining the representation of non-biomedical terms, this approach improves the clustering of related biomedical concepts, thereby enhancing the performance of embedding models in downstream tasks. This advancement has the potential to improve information retrieval, facilitate gene-disease association extraction, and support literature-based discovery by producing embeddings with greater semantic coherence.

Furthermore, the synonym replacement strategy holds promise for enhancing the construction of biomedical knowledge graphs, where accurate embeddings are essential for representing entities such as genes, proteins, and phenotypes. Improved embeddings can refine node representations and lead to more accurate predictions of relationships between biomedical entities, contributing to the advancement of computational biology and biomedical research.

In conclusion, the methodology presented in this study offers a scalable and effective means of improving biomedical embeddings through non-biomedical synonym replacement. This approach not only enhances the utility of existing embedding models but also provides a foundation for future work aimed at integrating similar strategies into more advanced architectures, further driving innovation in biomedical text analysis.
